# Determination of major and trace element variability in healthy human urine by ICP-QMS and specific gravity normalisation†

**DOI:** 10.1039/c8ra06794e

**Published:** 2018-11-13

**Authors:** Rebekah E. T. Moore, Mark Rehkämper, Katharina Kreissig, Stanislav Strekopytov, Fiona Larner

**Affiliations:** Department of Earth Science and Engineering, Imperial College London London SW7 2AZ UK r.moore13@imperial.ac.uk +44(0) 7974 779 418; Imaging and Analysis Centre, Natural History Museum London SW7 5BD UK; Department of Earth Sciences, University of Oxford Oxford OX1 3AN UK

## Abstract

Sixty five urine samples obtained during one or two non-consecutive days from 10 healthy individuals were analysed for major (Na, Mg, K, Ca) and trace (Co, Cu, Zn, As, Rb, Sr, Mo and Pb) element concentrations. Following microwave digestion, the analyses were carried out using ICP-QMS (inductively coupled plasma quadrupole mass spectrometry) incorporating a collision/reaction cell. Repeat analyses of quality control samples show that the procedure produces unbiased results and is well suited for routine urinalysis of the investigated elements. Concentrations were normalised using specific gravity (SG) and the resultant decrease in variability supports previous conclusions that SG-normalisation appropriately corrects for differences in urine dilution. The elemental concentrations of the individual urine samples show large differences in dispersion. Most variable are As, Co and Zn, with CVs (coefficients of variation) of >75%. The major elements as well as Rb, Sr and Mo display intermediate variability, whilst Cu and Pb have the least elemental dispersion with CV values of about 30%. A detailed assessment shows that the overall elemental variability is governed both by differences between individuals and variations for a single individual over time. Spot urine samples exhibit elemental concentrations that, on average, resemble the daily mean values to within about 30% for all elements except K and Rb. Diet-related changes in urinary element concentration are most prominent for Mg, K, Co, Rb and Pb. The concentrations of Co, As and Rb appear to vary systematically with gender but this may primarily reflect co-variance with specific diets.

## Abstract 2

Urinary element concentrations were quantified by ICP-QMS and variations over time, between individuals and with gender and diet were assessed.

## Graphical abstract

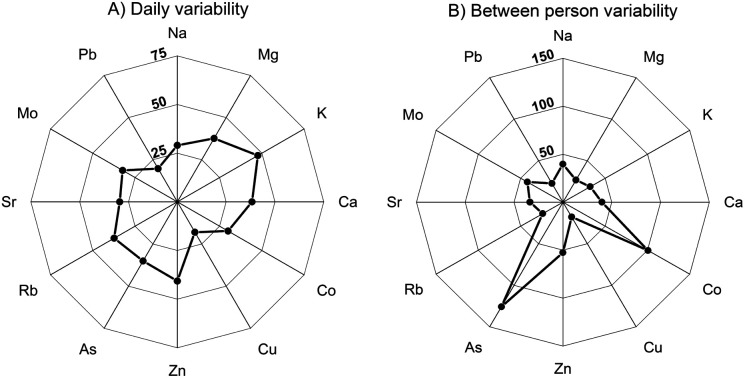

## 1. Introduction

A large number of chemical elements, often at specific concentrations or in specific oxidation states, are critical for normal functioning of the body.^1^ For example, Fe is a vital component of haemoglobin, which distributes oxygen within the body, and hundreds of enzymes contain Zn-specific binding sites.^2^ Maintaining the correct balance of essential elements is vital, and imbalances are linked with illness and disease.^3,4^ In biology, interactions between different elements, as well as between elements, organic molecules and tissues are intricate and many.^5–7^

Urine, a product of constant-rate kidney filtration of blood, represents a mixture of water-soluble waste metabolites. The study of urine (urinalysis) is well established and has been used to investigate exposure to hazardous elements or chemicals,^8–12^ to identify abnormalities in absorption,^13–17^ or for the study of diseases^18–22^ to determine causes and improve prognosis. Compared to analyses of blood and faeces, urine analyses are relatively straightforward, as sample pre-treatment is facilitated by the relatively small amounts of organic and inorganic solutes that are present. Urine sample collection is, furthermore, both non-invasive and easy to accomplish without the assistance of trained clinicians. The ‘cleanliness’ of collection is, however, an important factor to consider, particularly because sample vials contain small amounts of trace elements, which may be leached by the urine samples on contact and may suffice to bias concentration data for some elements. Sample collection must hence either rely on specially purchased ‘trace metal free’ containers or employ vials that are appropriately cleaned prior to use.

In the following, we present an investigation into the variability of elemental concentrations in healthy human urine, as determined by inductively coupled plasma quadrupole mass spectrometry (ICP-QMS). While sector field inductively coupled plasma mass spectrometry (SF-ICP-MS) was initially preferred for trace element analyses of biological samples,^23,24^ advances in technology, such as the perfection of collision/reaction cells, have led to significant improvements in data quality for trace element analyses of urine by low resolution ICP-QMS.^25–29^ The latter technique is employed in this study and the elements monitored encompass both major elements (Na, K, Mg, Ca) and a range of trace elements (Co, Cu, Zn, As, Rb, Sr, Mo and Pb).

The dietary sources of these elements are known (see ESI.1 Table S1†) and the effect of diet on the urinary concentrations of the elements has been investigated to an extent, but more research in this area is desirable. Associations between the consumption of caffeinated or alcoholic beverages and smoking with trace element metabolism and excretions have also been studied. For instance, urinary concentrations of Na, Mg and Ca may be increased by caffeine and alcohol consumption^30,31^ and alcohol consumption may increase Cu, Zn and As,^32–35^ whilst smoking has been linked with higher Cu, Zn, Sr and Mo^36^ in urine.

A number of previous studies have established reference ranges for the major and trace element concentrations of urine from healthy individuals.^23,25,26,29,37,38^ Generally, these investigations focus on differences between individuals from specific populations and some consider additional criteria, such as gender and smoking status. In contrast, the current study utilises multiple samples from individual participants to investigate the variability of urinary element concentrations during the course of a day and between days, as well as taking into account gender and diet. As such, the data collected here can help to establish whether first-void (spot) urine samples are able to provide a reasonable characterisation for the average daily urinary concentrations of various elements. In comparison with the standard practise of 24 hour sample collection, this would limit the sampling time for the patient, reduce sample storage space (and therefore cost), and lessen the risk of contamination by reducing patient–container interactions. This is particularly poignant for biologically active elements such as Zn, whereby the ubiquitous nature of the element in the environment renders contamination a key obstacle in establishing accurate records.^39^

## 2. Materials and methods

### 2.1. Urine sample collection and quality control materials

Ethical approval for the study was granted by the Imperial College Research Ethics Committee (ICREC reference 15IC3042). Participants were recruited *via* blanket email, whereby the following exclusion criteria were applied: (i) bacterial or viral infection, or any disease at the time of sample collection; (ii) history of kidney or urinary tract infections, or urethral syndrome, (iii) use of any medication or a hormonal intrauterine device; (iv) pregnancy; (v) in day 1 to 8 of the menstrual cycle at time of sample collection; (vi) under 18 years of age.

After giving informed consent, eligible volunteers were provided with 50 ml VWR metal-free centrifuge tubes (catalogue number 525-0463H, marketed as “certified free of major critical trace metals to less than 1 ppb”), a questionnaire and a food diary. Participants were given instructions to: (i) provide three to five mid-stream samples of urine over one or two non-consecutive 24 hour period(s), which were to include the first void of the day; (ii) fill in the questionnaire, to provide information on age, gender and smoking status; and (iii) complete a food diary for each day of sample collection, which informs on the intake of food, drink and any supplements. The exclusion criteria and sample collection technique are important to ensure that only samples from ‘healthy’ individuals were collected and to minimise element additions from other body tissues, medication or man-made appliances.

The analytical methods employed here were validated using three quality control materials. Seronorm™ Trace Elements Urine L-1 (Lot 1403080)^40^ is ideally suited for this purpose as it is similar in matrix and elemental concentrations to the samples analysed. Although a recommended material for quality control in toxicological analyses,^41^ the assigned and approximate reference concentrations of this material are, however, based on results from only a single laboratory. As such, two additional biological reference materials (RMs) that are certified for a number of elements were also employed for quality control: bovine muscle ERM-BB184 ^42^ and human blood serum BCR-639,^43^ both from the Institute of Reference Materials and Measurements.

### 2.2. Sample preparation

Samples were collected anonymously from participants and subsequently stored in a laboratory fridge, at 5 °C. Blank contributions from the VWR sample storage tubes were thereby minimised by processing samples soon after collection. All further sample preparation prior to the elemental analyses was performed in an ISO Class 6 metal-free laboratory equipped with Class 4 laminar flow hoods, in the Imperial College London MAGIC Research Centre. Distilled HNO_3_ (15.3 M, prepared in a Savillex™ DST-1000 acid purification system from an analytical grade acid), ultra-pure (18.2 MΩ cm) H_2_O and 30% ROMIL-UpA™ H_2_O_2_ were used throughout. Pre-cleaned Savillex™ fluoropolymer vials and acid-rinsed 15 ml polypropylene Sarstedt™ tubes were employed for sample storage throughout the preparation and measurement stages, respectively.

A 2 ml aliquot was pipetted out from each urine sample and mineralised by microwave digestion using an Ethos EZ system (Milestone S.r.l.), fitted with SK-10 high pressure rotor, in acid-cleaned 100 ml Teflon vessels. A blank was included with each set of 9 digestions. A 3 : 2 mixture of 15.3 M HNO_3_ and H_2_O_2_ up to a total of 7 ml was added to each sample and they were left inside a laminar flow hood to partially digest for about 12 hours. The vessels were then placed in the microwave oven, which was ramped up to a temperature of 210 °C over 60 minutes, and held there for 30 minutes to complete digestion. The sample digests were transferred into Savillex™ fluoropolymer vials, evaporated to dryness and the residues were re-dissolved in 1 ml 2 M HNO_3_. A 100 μl aliquot was then taken, dried and re-dissolved in 2 or 3 ml 0.1 M HNO_3_ (dilution factors of ×10 to ×15) for trace element analysis by ICP-QMS. A separate 10 μl aliquot was taken and diluted to 5 ml with 0.1 M HNO_3_ (dilution factor of ×250) for the determination of major element concentrations. Individual aliquots of the three quality control materials were digested and processed in the same manner as the urine samples.

### 2.3. Concentration measurements

#### 2.3.1. ICP-QMS

The major and trace element concentrations were determined at the Imaging and Analysis Centre of the Natural History Museum London, using an Agilent 7700x quadrupole mass spectrometer equipped with an octopole collision/reaction cell (CRC). The conditions of analysis, including parameters of the ICP-QMS, the different CRC gas modes (He, H_2_ and ‘no gas’) tested for the determination of major and trace element abundances and the choice of isotopes for each mode, are presented in ESI.1 Table S2.†

A series of trace element standard solutions, diluted using the same acid as the samples (0.1 M HNO_3_), and with concentrations of 1 ng ml^−1^, 5 ng ml^−1^, 10 ng ml^−1^, 50 ng ml^−1^ and 100 ng ml^−1^ were analysed at the beginning of each measurement session for calibration of the analyses, whereby Rh and In were added in-line at 1 ng ml^−1^ as internal standards. The 5 ng ml^−1^ standard solution was additionally run every 10 samples, to monitor instrumental drift. After each drift control measurement, a calibration blank was analysed to evaluate memory effects and cross contamination. Limits of quantification (LOQ) were calculated as 10 times the standard deviation of the calibration blank measured throughout the analytical run. For further quality control, a sample of Seronorm™ Trace Elements Urine L-1 was analysed at the beginning and end of each measurement session.

#### 2.3.2. Multiple collector ICP-MS (MC-ICP-MS)

The urinary Zn concentrations were also determined with the isotope dilution (ID) technique in measurements with a Nu Plasma HR multiple collector (MC) ICP-MS instrument.^44^ Such analyses are more robust and offer improved data quality compared to ICP-QMS.^45^ Further 10% aliquots of the urine sample digests were employed for this analysis. A ^64^Zn–^67^Zn double spike was added to and equilibrated with this aliquot and Zn was then isolated from the sample matrix by anion exchange chromatography. Additional analyses of bovine muscle ERM-BB184 and human blood serum BCR-639 were carried out for quality control of the ID-MC-ICP-MS technique. This procedure was employed as these two materials have very well-characterized Zn reference values and considering that the exact nature of the matrix is not critical, given that Zn is determined following anion exchange separation.

### 2.4. Blank correction and normalisation of concentration data

All results were corrected for the corresponding procedural blank, which contributed less than 20 ng ml^−1^ for Na, Mg, K and Ca, less than 1 ng ml^−1^ for Zn and Rb, less than 100 pg ml^−1^ for Cu and As, and less than 10 pg ml^−1^ for Co, Sr, Mo and Pb.

The specific gravity (SG) of each sample was measured using a 20 μl aliquot of urine with a Kern ORA-P Analogue Urine Refractometer, after samples were left to equilibrate to 20 °C. The SG data were then used to correct the measured elemental concentrations for the effects of varying flow rate, hydration status and total urine volume.^46^ Although creatinine normalisation is more frequently applied to urine solute concentrations,^12,19,20,24,29,46–49^ it was recently argued that an SG-based normalisation has a number of advantages, especially when comparing individuals with large differences in muscle mass or meat intake,^50^ and that it should hence be more widely adopted.^51,52^

The SG of ‘healthy’ urine falls between 1.00 and 1.03. It was shown that the SG for non-pathological samples, containing no haemoglobin, ketones, proteins, glucose and other substances not usually found in ‘healthy’ urine, correlates well with osmolality (*R*^2^ = 0.83).^53^ The method thus enables the identification of samples that are contaminated with other body tissues, as this is typically associated with urinary SG values of >1.03. The SG-normalisation of the concentration data applied the following equation:1
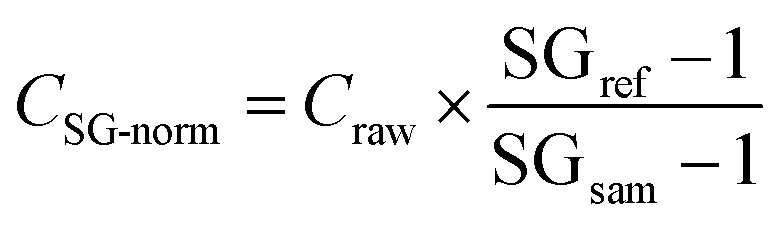
where *C*_raw_ is the measured element concentration, SG_sam_ is the specific gravity of the urine sample and SG_ref_ is a reference value. Here, the commonly used reference value of SG_ref_ = 1.02 was used because it represents the mean of ‘healthy’ human urine.^18,50–55^

## 3. Results

### 3.1. Participants and samples

A total of 65 urine samples were provided by ten volunteers, of which six were women and four men. The participants were between 23 and 33 years old, and all live and work in southeast England. Of these ten, six gave urine samples on two non-consecutive days and four gave samples on only one day. The questionnaires revealed that the samples are representative of five diet types (Table 1): omnivorous (all food), omnivorous but dairy free, pesco-vegetarian (all food, including fish and dairy but no meat), vegetarian (all food, including dairy, but no fish or meat) and vegan (all food except fish, meat or dairy). The cohort furthermore encompassed participants with varied alcohol and caffeine consumption as well as smoking habits (Table 1).

**Table 1:** Summary of information on the study participants P1 to P10

Participant	P1	P2	P3	P4	P5	P6	P7	P8	P9	P10
No. of samples provided	8	6	5	9	4	8	9	4	7	5
Sampling period (days)	2	2	1	2	1	2	2	1	2	1
Gender	Fem	Fem	Fem	Fem	Fem	Fem	Male	Male	Male	Male
Diet type^a^	Omv	Vgt	Psv	Omv	Veg	Daf	Omv	Omv	Omv	Vgt
Diet group^b^	Omv	No-m	No-m	Omv	No-m, no-d	No-d	Omv	Omv	Omv	No-m
Smoking status^c^	Never	Occs	Never	Never	Curr	Past	Past	Never	Never	Never
Alcohol intake^d^ (units per day)	<1	≥3	<1	<1	<1	1–2	<1	<1	1–2	≥3
Caffeine intake^e^ (drinks per day)	≥3	1–2	1–2	<1	≥3	≥3	<1	≥3	≥3	1–2

a Diets: omv – omnivorous; daf – dairy-free; psv – pesco-vegetarian; vgt – vegetarian; veg – vegan.

b Diet groups: no-m = no-meat – psv + vgt + veg; no-d = no-dairy – daf + veg.

c Smoking status: curr – current; occs – occasional.

d Alcohol intake is quantified in UK Alcohol units.

e Caffeine drinks include coffee and caffeinated soft drinks.

Three samples had SG values that exceeded the generally accepted upper limit of 1.03 for ‘healthy’ urine but as these results, between 1.031 and 1.035, were only marginally higher than the limit, the samples were kept in the dataset.

### 3.2. Quality control results

**Fig. 1:**
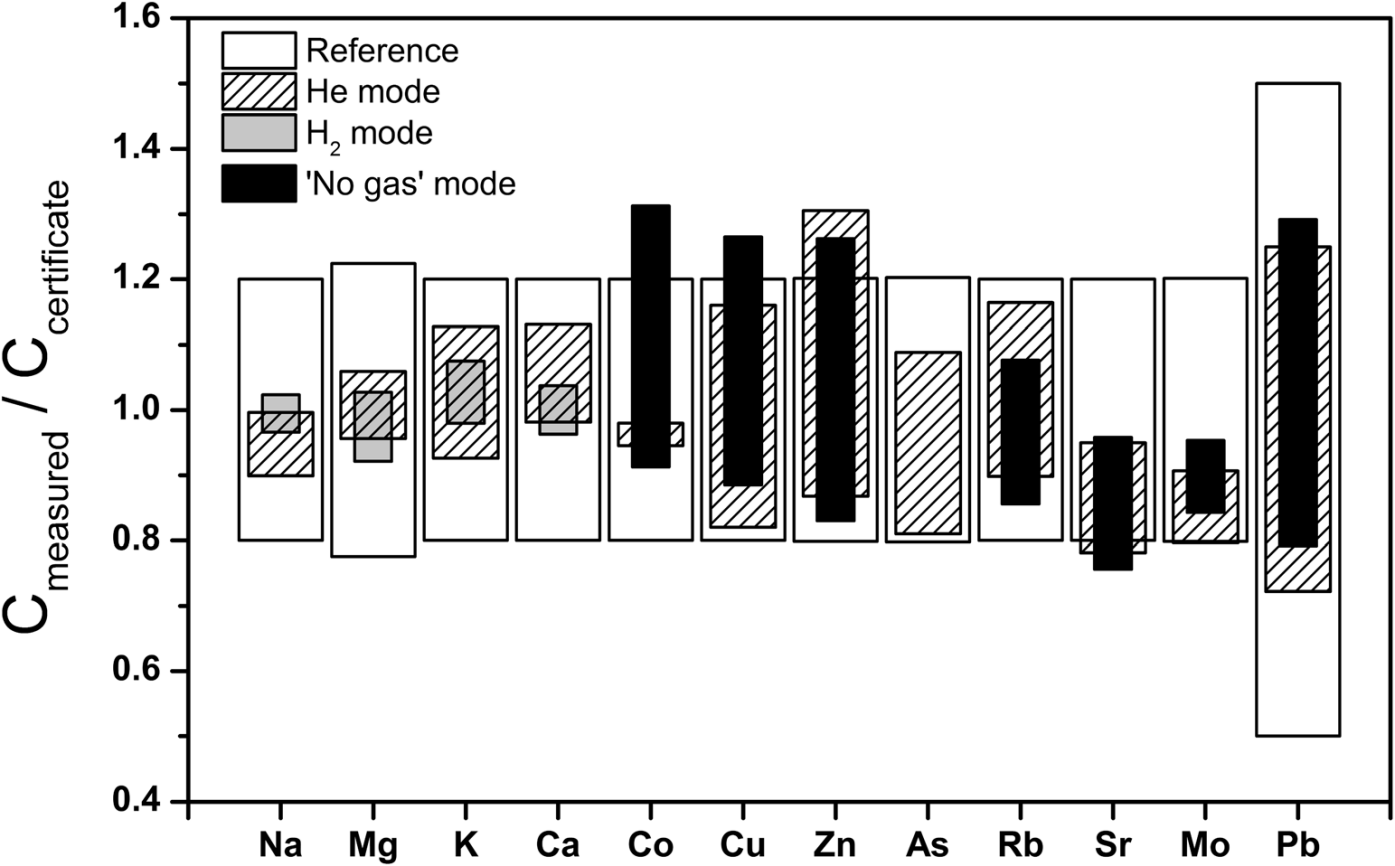
Major and trace element concentrations determined for Trace Elements Urine L-1 compared to reference values.

Repeat analyses of the Seronorm™ Trace Elements Urine L1 quality control sample by ICP-QMS were carried out to evaluate the performance of different gas modes for the determination of both major and trace elements (Fig. 1 and ESI.1 Table S3†). The major element measurements in He and H_2_ mode produced statistically indistinguishable results that are also in excellent accord with the reference values. Notably, whilst use of H_2_ in the CRC is often preferred for the determination of Ca abundances (as the major ^40^Ca isotope can be used due to the effective reduction of ^40^Ar^+^ interferences^56,57^), urinary Ca contents are high enough to allow accurate quantification of ^44^Ca when He is used as collision gas. The only instance where He mode did not show good agreement with certified values was for Ca in bovine muscle ERM-BB184 (ESI.1 Table S4†).

The ICP-QMS trace element analyses of the Seronorm™ Trace Elements Urine were conducted in both ‘no gas’ and He mode, whereby only the latter is useful for As due to molecular interferences from ^40^Ar^35^Cl^+^. As for the major elements, the results produced with the two gas modes are identical to one another and the reference values, within the quoted errors (Fig. 1 and ESI.1 Table S3†). It is conceivable that this good agreement with the SF-ICP-MS trace element reference values is at least in part due to the complete mineralisation of the sample *via* microwave-assisted digestion. This procedure, which was required in any case to enable Zn measurements by ID-MC-ICP-MS, helps to drastically reduce carbon-based molecular interferences^58,59^ from the urine matrix. Both gas modes furthermore generated results that agree well with reference data for Cu and Zn in bovine muscle ERM-BB184 but not for Zn in human blood serum BCR-639. In contrast, the Zn concentrations that were determined by ID-MC-ICP-MS are in full accord with the certified values for both quality control samples (ESI.1 Table S4†).

Based on these results, the major and the trace element analyses of the urine samples were conducted in H_2_ and He mode, respectively. For Zn, the concentration data that were produced with the more robust ID-MC-ICP-MS technique are further evaluated in the following. However, the urinary Zn data obtained by ICP-QMS with He in the CRC are also of good quality. This is demonstrated by the observation that the Zn concentrations obtained for the urine samples with the two instrumental methods differed by only about 10%, on average.

### 3.3. Sample results

In the following, the individual measured and SG-normalised urinary element concentrations are compared with one another and with published literature data (Table 2; ESI.2†). Overall, the mean, geometric mean, median and 95th percentile values calculated from the non-normalised elemental data are typically about 30 to 50% lower compared to the results for the SG-normalised concentrations. As such, this difference primarily reflects the SG-normalisation value of 1.020, which was also used in numerous previous investigations.^18,52,54,55^ Notably, the SG-normalisation clearly reduces the dispersion of the individual results. In detail, the coefficients of variation (CV) for all elements, except As, are lower by between 10 to more than 50% for the SG-normalised compared to the non-normalised data (Table 2). A similar observation also applies to the within-day dispersion of the urinary element concentrations for each individual (not shown). As such, the results support the previously stated conclusion that SG-normalisation provides a useful correction of elemental data for normal variations in urine dilution.^50–52^ Given this, the SG-normalised urinary element concentrations are further discussed in the following.

**Table 2:** Evaluation of the non-normalised and SG-normalised urinary elemental data for the individual samples of this study and comparison with (non-normalised) literature data^a^

		Na, μg ml^−1^	Mg, μg ml^−1^	K, μg ml^−1^	Ca, μg ml^−1^	Co, ng ml^−1^	Cu, ng ml^−1^	Zn, ng ml^−1^	As, ng ml^−1^	Rb, ng ml^−1^	Sr, ng ml^−1^	Mo, ng ml^−1^	Pb, ng ml^−1^
**Raw element concentrations**													
This study	Mean	2357	78	2760	114	0.42	8.67	305	33.3	1390	123	38.0	0.50
This study	Geom. mean	1928	57	2244	74	0.22	7.15	175	12.7	1119	92	28.0	0.42
This study	Median	2104	71	2569	76	0.20	7.20	184	12.5	1266	97	28.9	0.45
This study	95P	5078	191	6780	309	1.95	18.90	1424	206.5	3366	311	121.5	1.09
This study	SD	1334	55	1811	98	0.58	5.47	392	58.8	890	93	31.1	0.28
This study	CV (%)	57	70	66	86	138	63	129	176	64	76	82	57

**SG-normalised element concentrations**													
This study	Mean	3474	105	4052	151	0.54	11.4	341	44.4	1907	162	53.1	0.67
This study	Geom. mean	2971	87	3437	114	0.35	11.0	267	14.9	1724	142	43.2	0.64
This study	Median	3364	107	3302	139	0.25	10.6	286	11.6	1688	149	46.5	0.63
This study	95P	6891	185	8322	296	1.88	16.6	804	165.6	3503	296	117.1	1.12
This study	SD	1761	53	2397	89	0.59	3.0	263	78.1	884	77	32.2	0.21
This study	CV (%)	51	50	59	59	108	26	77	176	46	48	61	32

**Literature data (un-normalised element concentrations)**													
UK^c^^,^^d^	Ref. ranges^b^	460–6300	30–260	590–6350	50–380								
Germany^26^	Mean					0.387	9	269	34	1204	166	38	0.8
Germany^26^	Geom. mean					0.18	8	207	13	1071	132	26	0.5
UK^29^	Median					0.22	8.75	180	10.48	1090	80	29.13	0.47
Belgium^29^	Median					0.184	8.18	256	2.17			31.3	0.872
UK^29^	95P					1.04	19.33	730	152.4	2700	350	107.3	7.63
Germany^26^	95P					1.53	13	692	143	2424	444	94	2.1
Belgium^38^	97.5P					1.281	23.9	1432	13.2			135	3.45
Canada^37^	95P						25	1100	27			170	1.9

a 95P = 95th percentile; SD = standard deviation; CV (%) = 100× (SD/*x̄*) where *x̄* denotes the mean.

b Reference ranges calculated from reference values for 24 hour urine collection assuming excretion of 0.8 to 2.0 l of urine per day.

c Additional data source: S. Fuggle, Clinical biochemistry reference range handbook, East Sussex Healthcare, NHS Trust. 2018, 1.7, 1-17.

d Additional data source: Clinical biochemistry handbook, Ipswich Hospital NHS Trust. 2017.

A comparison of the results obtained in this study with published non-normalised urinary elemental data supports the statement that the samples investigated here were obtained from healthy individuals. In particular, for Na, Mg, K and Ca, both our measured and the SG-normalised results are in good accord with the published reference ranges of UK Hospitals (Table 2), with only a few individual samples showing relatively minor deviations. For trace elements (Co, Cu, Zn, As, Rb, Sr, Mo and Pb), the new results also provide mean, geometric mean and median values that are in good agreement with recent reference ranges from studies conducted in the UK,^29^ Germany,^26^ Belgium^38^ and Canada.^37^ Unsurprisingly, our non-normalised results show better agreement with the non-normalised reference ranges than the SG-normalised concentrations, which are generally somewhat higher. For Sr, however, the SG-normalised results of this study show better agreement with the published reference values (Table 2).

Considering the overall variability of our new concentration data, the elements fall into four groups. Arsenic and Co thereby have the most variable concentrations, with CV values of >100%, whilst Zn is still fairly variable with a CV of about 75% (Table 2). The large group of elements with intermediate variability, and CVs of about 45 to 60%, includes the major elements (Na, Mg, K and Ca) as well as Rb, Sr, and Mo. Copper and Pb have least variable element concentration found in this study, with CV values of about 25 to 30% (Table 2).

## 4. Discussion

### 4.1. Evaluation of relationships between elements

Correlations between elements were investigated using the results obtained for individual samples and evaluated using *r* and *r*^2^ values (see ESI.1 Table S5†). The two most significant correlations, for K–Rb (*r*^2^ = 0.81) and Ca–Sr (*r*^2^ = 0.66), are not unexpected as the element pairs are neighbours from the same group of the periodic table that have similar chemical properties and ionic radii. The lighter elements, K and Ca, are major elements in the body, whilst Rb and Sr are trace elements, which can substitute for the former.^60^ Further element pairs that display correlations with *r*^2^ ≥ 0.16, which are still statistically significant at the 1% level, are Na–K with a positive co-variation (*r*^2^ = 0.23) as well as Co–Ca (*r*^2^ = 0.21) and Co–Zn (*r*^2^ = 0.16) with negative co-variations. Zinc clearly stands out amongst the elements studied here, in that it predominantly displays negative correlations with other elements, including Na, K, Co, Rb and Mo (ESI.1 Table S5†).

### 4.2. Variability of the results between different individuals

**Fig. 2:**
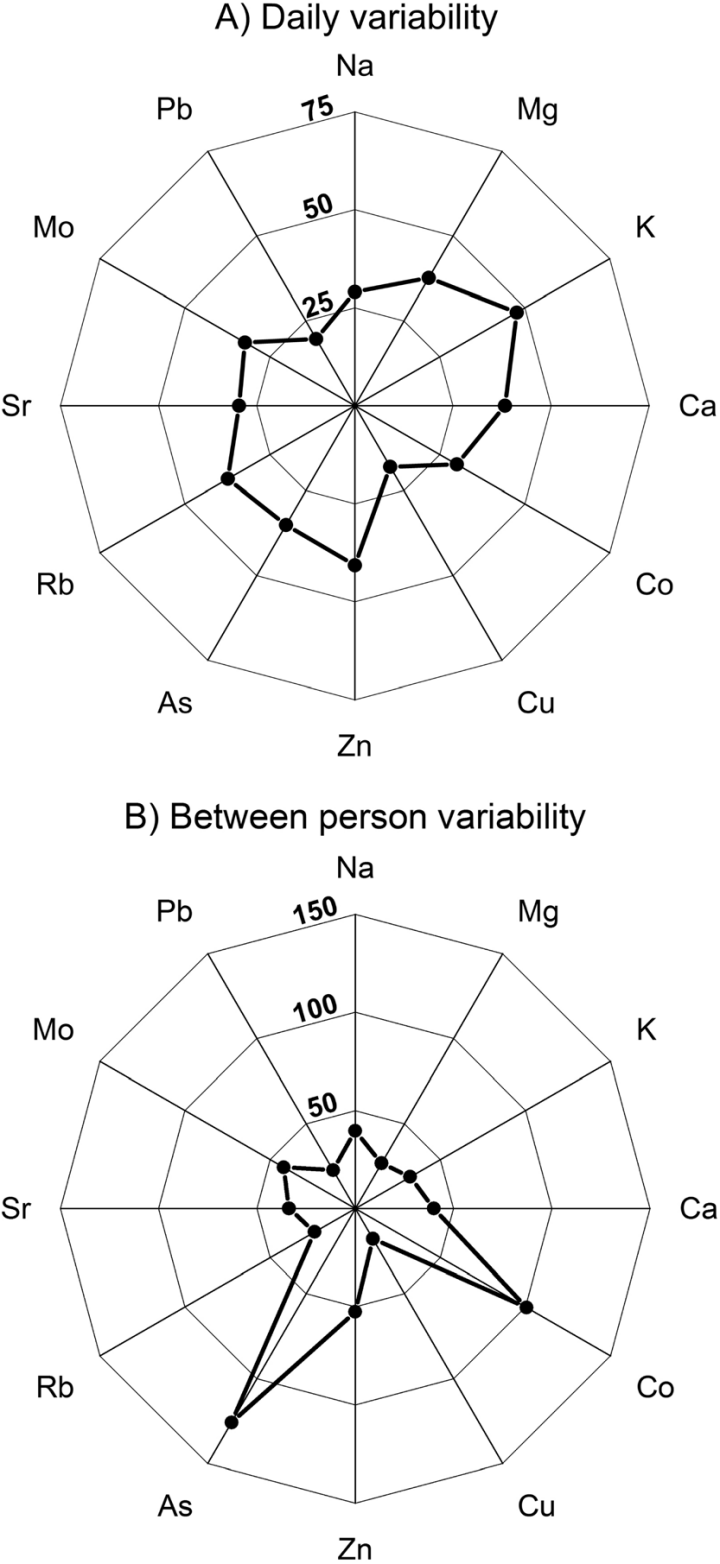
Average variability of major and trace element concentrations in urine of participants, defined by coefficients of variation (CV values in %), both within single days (A) and between participants (B).

In the following, the variability of the mean urinary elemental concentrations determined for the 10 participants is discussed, based on (i) the CV values defined by the data (Fig. 2 and Table 3), and (ii) by assessing differences between the mean values for each individual and the overall median (ESI.1 Table S6†). The median value is specifically used for this comparison because this is less biased by spurious high or low concentrations than the mean. For most elements, however, the overall mean and median differ by less than 10% (Table 3).

**Table 3:** Information on (i) averages and associated variability determined from the mean element concentrations of the 10 study participants; (ii) average elemental variability for individuals, determined from the elemental compositions of individual urine samples collected on a single day or two non-consecutive days; (iii) mean *z*-scores for the first void element concentrations relative to the daily mean concentrations^a^

Results	*n*	Na	Mg	K	Ca	Co	Cu	Zn	As	Rb	Sr	Mo	Pb
**Evaluation of differences/similarities between participants by considering mean results for each individual**													
Mean (μg ml^−1^, ng ml^−1^)	10	3678	107	4156	150	0.57	11.7	336	43.9	1919	165	55.8	0.70
Median (μg ml^−1^, ng ml^−1^)	10	3335	111	3911	148	0.33	11.4	309	15.7	2001	168	53.1	0.69
SD (μg ml^−1^, ng ml^−1^)	10	1457	29	1340	60	0.58	2.1	177	55.3	460	56	23.4	0.16
CV (%)	10	40	27	32	40	101	18	53	126	24	34	42	23

**Evaluation of variability for individuals over 1 Day or 2 non-consecutive days**													
1 Day CV (%)	16	29	38	48	38	30	18	41	35	37	29	32	20
2 Day CV (%)	6	40	41	52	45	32	17	49	67	40	34	48	23
2 Day CV/1 Day CV	1.4	1.1	1.1	1.2	1.1	0.9	1.2	1.9	1.1	1.2	1.5	1.2	

**Comparison of first-void concentrations to daily means**													
*z*-scores	16	0.9	0.8	1.6	0.8	0.9	0.8	1.0	0.9	1.4	0.9	0.8	0.8

a The concentrations of the major elements Na, Mg, K, Ca are given in μg ml^−1^; all other (trace) element contents are given in ng ml^−1^*n* = number of results in dataset. SD = standard deviation. CV (%) = 100× (SD/*x̄*) where *x̄* denotes the mean.

Overall, the mean participant data show somewhat less dispersion, and hence somewhat lower CV values, than the complete dataset of all individual results (Tables 2 and 3). There are, furthermore, some clear differences between the elements. The most constant levels are typical for Mg, Cu, Rb and Pb which display CV values of <30%, whereby less than half of the participants show individual deviations from the median of >25% (Table 3 and ESI.1 Table S6†). The concentrations of Na, K, Ca, Sr and Mo display moderate between-individual differences, with average CV values of 30 to 45%, whilst Zn is fairly variable with CVs of 45 to 55%. The elements with the most pronounced inter-individual differences in concentrations are Co and As, which both have CV values of more than 100%. The increased variation seen for these elements is expected, as participants will have been exposed to different levels of their ionic forms.^61,62^

### 4.3. Variability of results for single individuals over time

To assess the variability of urinary element concentration for different individuals, CV values were calculated from the element concentrations of the individual samples taken from each participant, both over a single day and, where available, over two non-consecutive days (Fig. 2, ESI.1 Tables S7a and b†). For each element, a mean CV value was determined, which characterises the average elemental variability over 1 Day and 2 Day periods (Table 3).

#### 4.3.1. Within-day variations in element concentrations

The 1 Day CV values that were calculated to assess element variability for a single day show that Cu and Pb have the most constant within-day concentrations, whereby most individual CV values are ≤25% and the mean CVs are <20% (Fig. 2, Table 3 and ESI.1 Table S7a†). Sodium, Co and Sr display moderate within-day variations, whereby about half of the 1 Day CV values are ≤50%, such that the average CVs are ≤30%. The concentrations of more highly variable elements (Mg, Ca, As, Rb and Mo) have mean CV values of 30 to 40% because the majority of individual results have CVs of >25%. The most highly variable elements are K and Zn, with overall CV values exceeding 40%, whereby all or nearly all individual days have dispersions characterised by CVs >25% (Table 4 and ESI.1 Table S7a†).

**Table 4:** Summary of data to evaluate relationships of urinary elemental concentrations with gender and diet regimes^a^

		Na	Mg	K	Ca	Co	Cu	Zn	As	Rb	Sr	Mo	Pb
**Evaluation of gender (male *versus* female)**													
Mean/mean	Male/fem	1.0	0.7	0.7	1.1	**0.2**	1.2	1.8	**0.1**	0.8	1.2	0.8	0.8
Median/median	Male/fem	1.1	0.8	0.7	1.0	**0.4**	1.4	1.7	**0.2**	0.8	1.2	1.1	0.9
*P*	Male *vs.* fem	0.91	0.09	0.07	0.67	**0.04**	0.11	0.08	**0.05**	**0.05**	0.29	0.55	0.26

**Evaluation of diet (no-meat and no-milk *versus* omnivorous)**													
Mean/mean-omv	No-meat	1.3	1.4	1.6	1.0	**2.5**	1.0	0.6	1.4	1.2	1.2	1.3	1.5
Median/median-omv	No-meat	1.6	1.6	1.7	1.1	**2.2**	1.0	0.7	**3.4**	1.2	1.4	1.6	1.6
*P* (*vs.* omv)	No-meat	0.24	**0.04**	**0.01**	0.79	0.10	0.82	0.15	0.66	0.19	0.17	0.37	**0.003**
Mean/mean-omv	No-dairy	1.0	1.4	1.5	**0.3**	**6.4**	1.0	**0.3**	**0.4**	1.4	0.7	1.0	1.2
Median/median-omv	No-dairy	0.8	1.3	1.3	**0.1**	**8.8**	0.9	**0.4**	1.1	1.2	**0.4**	0.8	1.1
*P* (*vs.* omv)	No-dairy	1.00	0.08	0.08	**0.01**	**3 × 10** ^**−7**^	0.78	0.06	0.50	**0.04**	0.30	0.95	0.20

a The *P* values were obtained using standard Student's *t*-test procedures. Results that may be indicative of significant differences between genders or diet groups are highlighted in bold.

#### 4.3.2. Day-to-day variations in elemental concentration for individuals

The data collected for six participants that provided samples on two non-consecutive days are evaluated to determine 2 Day CV values. With the exception of Cu, these are typically about 10 to 20% larger than the 1 Day CVs but the relative variability of the elements is largely similar (Table 3 and ESI.1 Table S8b†). In detail, Cu and Pb are also the least variable elements for the 2 Day sampling period, with Co, Sr showing intermediate, Mg, Ca, Rb fair, and K, Zn high variability. This is supported by the ratio of the mean 2 Day to 1 Day CV values, where the above elements reveal ratios 0.9 to 1.2 (Table 3 and ESI.1 Table S8b†). In contrast, clear differences are evident for Na, Mo and As, as these have higher 2 Day CV/1 Day CV ratios of 1.4 (Na), 1.5 (Mo) and 1.9 (As). For the two former elements, the higher 2 Day variability in elemental concentrations is, however, predominantly caused by the unusually high 2 Day CV value of a single participant. Arsenic, in contrast, displays 2 Day CV values of >50% for all but one participant, due to large day-to-day variations in mean As concentrations for the individuals (Table 3 and ESI.1 Table S8b†).

#### 4.3.3. Comparison of between-individual and within-individual variability

Considering these results, the overall dispersion of elemental concentrations observed for the individual samples (Table 2) reflects elemental variability both between different individuals and for single individuals over time (Table 3). Notably, there are clear differences between elements concerning the significance of these two factors. For seven of the 13 elements, the elemental dispersion between individuals is roughly similar to the observed 1 Day and/or 2 Day variability. This group includes two major elements of intermediate variability (Na, Ca) as well as trace elements of high (Zn), intermediate (Sr, Mo) and low variability (Cu, Pb). A second group of elements, with intermediate variability, encompasses Mg, K and Rb. The concentrations of these three elements display significantly lower dispersion between different participants than within single individuals over time. The third group of elements, which encompasses Co and As, features more variable element concentrations for different individuals than in single individuals over 1 Day or 2 Day sampling periods (Table 3).

#### 4.3.4. Differences in concentration between first void samples and daily means

All participants are healthy young adults, so their kidney function should be normal and urination during sleeping hours is expected to be low. Therefore, relatively large amounts of the elements will be filtered through the kidneys before the bladder is emptied in the morning with the first void. The following analysis assesses how representative the first void elemental sample is for the daily mean urinary element concentrations of a given individual. To this end, a z-score was calculated,2
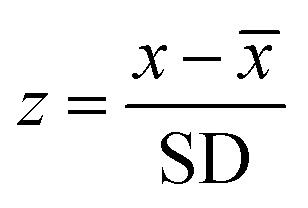
where *x* is a particular first void concentration, whilst *x̄* and SD are the concentration mean and standard deviation for the same day. For each element, an overall mean first-void *z*-score was determined from the 16 individual results (Table 3 and ESI.1 Table S8†).

Notably, the calculations reveal mean *z*-scores of less than 2 for all elements (Table 3) and there are only a few individual results with *z*-scores >2 (ESI.1 Table S6†). Overall, the calculations show that Mg, Ca, Cu, Mo and Pb have the lowest mean *z*-scores of between 0.7 and 0.8, whilst the highest values are recorded by K and Rb, with *z*-scores of 1.4 to 1.6. The remaining elements, Na, Co, Zn, As and Sr have intermediate *z*-values of between 0.9 and 1.0 (Table 3). Overall, these results confirm that spot urine samples can be used to obtain a reasonable approximation of the daily mean urinary elemental concentrations of an individual. This conclusion is confirmed by detailed analyses of the results, which show that there is no consistent positive or negative offset between data for spot samples and the daily mean concentrations. The exception is K, for which there is a clear daily diurnal variation for the majority of participants, whereby the concentration is notably higher in samples 2 and/or 3 of each day. This midday increase in K excretion was previously documented.^63–65^

### 4.4. Evaluation of factors that may impact urinary element concentrations

In the following, factors that may have an impact on the observed elemental variability are considered. Whilst information on smoking, caffeine and alcohol consumption habits were provided by the study participants, these data (see ESI.1 Tables S9a–c†) reveal no clear correlation with and hence impact on urinary element concentrations. However, this may reflect, at least in part, the limited size of the dataset. The following discussion therefore focuses on the impact of gender and diet on differences in urinary element concentrations.

#### 4.4.1. Correlations with gender

Of the ten participants, six were female and four were male. To assess possible impacts of gender on urinary element concentrations, mean and median female and male concentrations were determined from the mean daily results of both cohorts (ESI.1 Table S10†). These means and medians were then compared and differences between the populations were assessed using a standard Student's *t*-test, whereby *P* values of ≤0.05 were regarded as showing a significant difference related to gender (Table 4 and ESI.1 Table S10†). This comparison reveals significantly lower mean and median Co and As concentrations for males, such that populations differ at the *P* = 0.05 level. Whilst the mean and median Rb contents of males and females differ only by about 20%, the *t*-test also indicates a difference between the samples, with *P* = 0.05. Slightly higher *t*-test *P* values of between 0.07 and 0.09 were identified for Mg, K and Zn (Table 4). The CV values for the male and female data furthermore reveal element concentrations of similar variability, within a factor of about 2, for all elements save Co, which is significantly more variable for the female participants (ESI.1 Table S10†).

#### 4.4.2. Correlations with diet

As four of the five diet types into which the participants were originally grouped featured only one or two participants, the diet types were assigned to three diet groups: omnivorous (*n* = 5), no-meat, which includes pesco-vegetarian, vegetarian and vegan individuals (*n* = 4), and no-dairy, which includes dairy-free (but otherwise omnivorous) and vegan individuals (*n* = 2) (Table 1). For each diet group, mean and median urinary element concentrations were determined from the daily mean results of relevant individuals. The data for the no-meat and no-dairy group were then compared to the omnivorous group, using both the mean and median results as well as with a standard Student's *t*-test (Table 4 and ESI.1 Table S11†).

A number of observations suggest that diet has an impact on the element budget of urine. The mean and median urinary Co contents of the no-meat group are strongly elevated, whilst more moderate increases are observed for Mg, K and Pb. Based on the *t*-test values, using a cut-off value of *P* ≤ 0.05, the Mg, K and Pb concentrations of the no-meat group differ significantly from the omnivores (Table 4). In contrast, the *t*-test reveals no significant difference for the urinary Co concentrations of omnivores and no-meat participants, primarily because the Co contents of both populations are highly variable (ESI.1 Table S11†). In addition, the median As concentration of the no-meat group is much higher than the median value for the omnivores but this primarily reflects the high variability of both populations.

A comparison of the urinary element concentrations between the no-dairy group and omnivores also reveals some clear differences. In particular, the no-dairy individuals define much lower mean and median values for Ca and Zn, as well as somewhat lower results for Sr. Also obvious are the high mean and median urinary Co concentrations of the no-dairy group, which exceed the omnivore values by more than a factor of 6 (Table 4). In addition, somewhat higher Mg, K and Rb contents are observed for the urine of no-dairy participants than for omnivores. Based on the *t*-test *P* values, the differences between these two dietary groups are significant for Ca and Co, as well as Rb. Furthermore, relatively low *P* values of 0.06 to 0.08 were determined for Mg, K and Zn (Table 4).

### 4.5. Discussion of individual elements

In the following, the results for individual elements are discussed, in particular to differentiate between possible gender- and diet-related impacts on urinary concentrations. Unfortunately, this is not a straightforward exercise as the female cohort of this study features a more diverse range of diets than the males, whereby both individuals of the no-dairy group and three of the four no-meat participants are female (Table 1).

#### 4.5.1. Major elements

Major elements (Na, Mg, K and Ca) show intermediate variability in urine, for the overall dataset as well as between individuals and over time (Tables 2 and 3). Renal Na handling is responsible for maintaining whole body plasma volume, and as such it is tightly regulated, whereby ≤1% of total plasma Na is normally excreted. For Na, the urinary concentrations in this study of healthy participants show no relationship with gender or diet, echoing recent findings.^66,67^ In contrast, no-meat individuals clearly have higher urinary Mg, K concentrations, whilst no-dairy individuals feature low urinary Ca (Table 4 and ESI.1 Table S11†). In fact, Participant 6 with a dairy-free diet, has daily mean urinary Ca contents that are about a factor of 10 lower compared to the overall mean value. The low urinary Ca concentrations most likely reflect the lack of dairy products rich in Ca and vitamin D in the dairy-free and vegan diets, despite fortification of some dairy substitutes with both of these nutrients (ESI.1 Table S1†). The higher fibre content of a vegan diet may also contribute, due to the inhibitory effect fibre has on Ca gut absorption.^68^ As urinary Mg and K concentrations are also thought to be governed, at least in part, by diet,^69,70^ the higher Mg and K levels of samples from no-meat participants may be related to the increased consumption of foods with high Mg and K contents, including some vegetables, nuts, seeds and fish by individuals that do not consume meat (ESI.1 Table S1†).

#### 4.5.2. Trace elements

Cobalt and As display the highest dispersion in the concentration data (Table 2), primarily due to high variability between different individuals (Table 3). For single individuals, urinary Co contents show relatively little variation, whilst As is the most variable element for 2 day periods (Table 3). The urinary Co contents vary systematically with gender (whereby females have higher Co) and diet, as individuals from the no-dairy group have significantly higher urinary Co than the omnivores (Table 4). As a consequence, the data also reveal a statistically significant negative correlation between urinary Ca and Co concentrations (*r*^2^ = 0.20; ESI.1 Table S4†). Given that both individuals of the no-dairy cohort are female, it is unclear, however, which of these factors actually impacts the urinary Co contents. A recent study also found higher Co concentration in 24 hour urine that had undergone creatinine normalisation, but the opposite was seen for concentrations that were not normalised.^48^

Urinary and blood Co concentrations are utilised as a biomarker for recent dietary or environmental exposure to this metal.^61^ Notably, Co is a key constituent of vitamin B12, which is involved in the synthesis of haemoglobin. Furthermore, serum ferritin and Co concentrations were previously reported to define a negative correlation.^71^ These observations are indicative of links between Co and Fe homeostasis. As such, differences in urinary Co concentrations might reflect the iron status of the body^17^ and thus gender-related differences in Fe metabolism. However, the current study lacks male individuals in the no-dairy cohort to further test this hypothesis. Alternatively, the observed dispersion of urinary Co concentrations may reflect preferential consumption of foods that are either naturally rich in Co, such as leafy vegetables, cereals, seeds and fish or the presence of varying levels of vitamin B12 in the dairy-free diet (ESI.1 Table S1†).^72^ Non-dairy cohorts are subject to reduced vitamin B12 intake through a lack of dairy milk products, but may also be compensated due to vitamin B12 fortification of many substitute foods, such as soya milk. The proportion from vitamin B12 of the overall Co dietary intake is small,^73^ therefore it is hard to predict if, on its own, this would be an impactful contributor to urinary Co concentrations. Further urine analyses of additional individuals with varied diets, and in particular of males with no-dairy diets, and targeted dietary analyses will be able to shed more light on this issue.

The origin of the high variability of urinary As contents is also unclear from the current results. Whilst the data suggest that females have systematically higher As concentrations than males, the observation that urinary As varies significantly within individuals over longer periods of time hints at a relationship unrelated to gender. Previous studies have highlighted that seafood consumption is typically associated with high urinary As levels (ESI.1 Table S1†).^35,37^ It is hence possible that the three very high As concentrations observed here, of about 125 to 185 ng ml^−1^ for the pesco-vegetarian and two omnivorous participants, reflect short-term maxima that were generated by the consumption of fish or other foods, which are high in As. Consumption of fish on the day of urine collection is confirmed by the pesco-vegetarian participant in their food diary.

Zinc also shows urinary concentrations that are fairly variable overall (Table 2), whereby the concentrations show considerable dispersion both between individuals and for single participants over time (Table 3). Diet has a known influence on Zn mineral balance and in developed countries dietary choices are responsible for differences seen between groups.^74^ Vegetarian and vegan diets are lacking in Zn-rich meat and fish, but plentiful in phytates and fibre, which decrease the bioavailability of any Zn intake (ESI.1 Table S1†).^75–78^ Our data suggest that males have higher urinary Zn than females. Gender bias with dietary choices within diet groups is most likely responsible for the gender effect seen here, although this has been previously documented in a 24 hour urine study.^48^ For example, even within an omnivore group, females typically consume more phytate containing products (*e.g.*, salad) alongside meat, leading to a reduced Zn absorption for females not seen in males.^79^ The no-dairy group features the lowest Zn urinary content. This is likely to be dominated by the phytate influence in the diet rather than the lack of dairy, as casein, a major milk protein, is known to inhibit Zn absorption.^77^ Although there has been no consensus in recent literature, part of the within-day variation may also be due to diurnal cycles, similar to those seen in K concentrations.^80–84^

Similar to Zn, concentrations of Mo are also fairly variable in individual samples, with intermediate variability both between and over time for single study participants (Tables 2 and 3). The cause of this dispersion remains unclear at present, as neither gender nor diet show any significant relationship with the Mo contents (Table 4).

Rubidium and Sr also display intermediate variability. The former displays more variability within single individuals over time than between different participants (Table 3). Whilst this may imply that the possible link between urinary Rb levels and gender (Table 4) is an artefact of the small dataset, lower Rb contents in male urine have been observed previously.^9^ The results of the current study suggest, however, that the observed dispersion of Rb concentrations is most likely tied to diet. This conclusion is supported by the strong co-variation of Rb with K (ESI.2†) and the observation that both elements are somewhat enriched in the urine of the no-meat and no-dairy groups (Table 4), possibly due to enhanced intake of foodstuffs rich in alkali elements, such as vegetables (ESI.1 Table S1†). Similarly, the strong correlation of urinary Ca and Sr concentrations hints at a control of Sr contents through diet, *via* the consumption of dairy products. Whilst this is not clearly reflected in a direct comparison of the no-dairy and omnivore diet groups (Table 4), this may be due to the small size of the former cohort (Table 1).

Copper and Pb have the least variable urinary concentrations of the elements investigated here, whereby the dispersion is small both between individuals and for single participants over time (Tables 2 and 3). For Cu, this observation is in accord with previous findings^29^ whereby the Cu levels show no clear relationship with gender or diet. Bodily regulation of Cu is maintained through faecal rather than urinary excretion. The extremely small proportion lost *via* the renal system is negligible when total Cu excretion is considered,^85^ therefore the findings here are to be expected. In contrast, the Pb concentrations do appear to be linked to diet, whereby the no-meat participants have significantly higher Pb than the omnivores (Table 4). Given the pervasive contamination of the environment with anthropogenic Pb, even after the use of leaded gasoline was phased out, it is conceivable the relatively constant urinary Pb levels are a consequence of a relatively constant and high anthropogenic Pb background. Of further interest is the observation that the Pb contents are significantly higher for individuals of the no-meat diet group. This might reflect higher intake of Pb from the consumption of food, such as vegetables, that are particularly affected by Pb pollution.

## 5. Conclusions

A total of 65 urine samples were collected for this study on one or two non-consecutive days from 10 healthy individuals. Following microwave-assisted digestion, the samples were analysed for both major element (Na, Mg, K, Ca) and a range of trace element (Co, Cu, Zn, As, Rb, Sr, Mo, and Pb) concentrations by ICP-CRC-QMS in using a CRC in H_2_ and He mode, respectively. Repeat analyses of quality control samples demonstrate that at the concentrations observed in urine, He mode measurements produce unbiased results for all investigated elements and thus constitute a straightforward and robust method for routine urinalysis.

The concentration data display significantly lower dispersion following normalisation of the results to a common specific gravity (SG). This observation supports previous studies, which showed that SG-normalisation provides a useful correction of element concentrations for differences in urine dilution that can be used instead of normalisation to the creatinine content.^50–52^ We recommend SG-normalisation of major and trace element concentrations in urine in similar studies and in the clinical setting, due to the simplicity of the method, low running costs and portability, compared to the traditional procedure of creatinine normalisation.

The concentration data that were obtained for the individual urine samples reveal large differences in elemental dispersion. Arsenic, Co and to a lesser extent Zn have the most variable concentrations, with CV values of >75%. Major elements as well as Rb, Sr and Mo display intermediate variability (with CV ≈ 45–60%), whilst Cu and Pb show the least elemental dispersion with CVs of about 25 to 30%. Detailed evaluation of these results demonstrates that the elemental variability reflects both differences between individuals and changes in concentrations observed for a single individual over time. The relative importance of these two factors for the overall dispersion of the data differs significantly between elements. Despite the observed temporal variability of urinary element levels, the first void urine samples were found to have elemental concentrations, which resemble the daily mean values to within 30 to 60% for K, and Rb, and to within 20% or lower for all other elements. As such, spot urine samples appear to provide a reasonable approximation for daily mean urinary element concentrations for the elements studied here.

An assessment of whether urinary elements concentrations have any systematic relationships with gender and/or diet provides a number of significant observations. In detail, the results indicate that impacts of diet on urinary element levels are most likely for Mg, K, Co, Rb and Pb. Furthermore, Co, As and Rb appear to vary systematically with gender, but this observation may be a consequence of the investigated cohort, as the participants harbour a correlation between gender and some diet groups. As such, further urine samples from additional individuals are needed to better constrain whether the variability of the latter elements is related to gender or diet.

## Conflicts of interest

There are no conflicts to declare.

## Supplementary Material

RA-008-C8RA06794E-s001

RA-008-C8RA06794E-s002
