# Supplementary material for: Determination of major and trace element variability in healthy human urine by ICP-QMS and specific gravity normalisation
Source: RSC Adv. 2018 Nov 13;8(66):38022–35. doi: 10.1039/c8ra06794e (PMC9089848; doi:10.1039/c8ra06794e)
Supplement: RA-008-C8RA06794E-s001 [file RA-008-C8RA06794E-s001.pdf]

Electronic Supplementary Material (ESI) for RSC Advances.
This journal is © The Royal Society of Chemistry 2018

## Supplementary Information (Part I) to:

# Determination of major and trace element variability in healthy human urine by ICP-QMS and specific gravity normalisation

**Moore RET · Rehkämper M · Kreissig K · Strekopytov S · Larner F**

R. E. T. Moore (✉) · M. Rehkämper · K. Kreissig
Department of Earth Science and Engineering
Imperial College London
London SW7 2AZ
UK
Email: r.moore13@imperial.ac.uk
Tel: 07974 779 418

S. Strekopytov
Imaging and Analysis Centre
Natural History Museum
London SW7 5BD
UK

F. Larner
Department of Earth Sciences
University of Oxford
Oxford OX1 3AN
UK

**Table S1:** Sources of major and trace elements measured in this study in commonly eaten food and drink.

| Element | Food source | Ref(s). |
|---|---|---|
| Na | Table salt, cured meat and fish, egg | 1, 2 |
| Mg | Seeds, cereals, nuts, spinach, chocolate, meat, dairy products | 1, 2, 3 |
| K | Nuts, dried fruit, root vegetables, tomato, pepper, leek, pulses, avocado, banana, chocolate, orange juice, milk, tea, coffee | 1, 2, 3 |
| Ca | Milk, yogurt, cheese, fish (e.g. salmon, sardines), tofu, vegetables (e.g. broccoli, turnip) | 1, 2 |
| Co | Meat, fish, shellfish, leafy vegetables, legumes, nuts, seeds | 2, 3 |
| Cu | Liver, cocoa powder, nuts, legumes, shellfish | 2,3, 4 |
| Zn | Meat, fish, shellfish, legumes, nuts, seeds, bread | 1, 2, 3 |
| As | Seafood, water, cereals | 2, 5, 6 |
| Rb | Asparagus, lettuce, carrot, black tea, coffee, cocoa | 7 |
| Sr | Seafood, gram, nuts, kale, tea | 8 |
| Mo | Legumes, leafy vegetables, liver, cereals, nuts, milk | 2, 4 |
| Pb | Water, milk, spinach, chocolate, cereal | 9, 10 |

**Table S2:** Operational conditions of ICP-QMS

| | 'No gas' mode | He mode | $H_2$ mode |
|---|---|---|---|
| RF power | 1550 W | | |
| Plasma gas flow | 15 l/min | | |
| Carrier gas flow | 1.03–1.07 l/min | | |
| Dilution gas flow | 0.00 l/min | | |
| CRC gas flow | – | 5 ml/min (He 99.9995%) | 6 ml/min ($H_2$ 99.9999%) |
| Octopole bias | –8.0 V | –18.0 V | –18.0 V |
| Cell entrance | –30 V | –40 V | –40 V |
| Cell exit | –50 V | –60 V | –60 V |
| Deflect | 13.2 V | 1.4 V | –0.4 V |
| Plate bias | –40 V | –60 V | –60 V |
| Nebuliser type | Micromist | | |
| *Data acquisition parameters* | | | |
| m/z isotopes measured | $^{59}$Co, $^{65}$Cu, $^{66}$Zn, $^{75}$As, $^{85}$Rb, $^{88}$Sr, $^{95}$Mo, $^{206}$Pb, $^{207}$Pb, $^{208}$Pb | $^{23}$Na, $^{24}$Mg, $^{39}$K, $^{44}$Ca, $^{59}$Co, $^{63}$Cu,$^{65}$Cu, $^{66}$Zn, $^{75}$As, $^{85}$Rb, $^{88}$Sr, $^{95}$Mo, $^{206}$Pb, $^{207}$Pb, $^{208}$Pb | $^{23}$Na, $^{24}$Mg, $^{39}$K, $^{40}$Ca |
| m/z internal standards | $^{103}$Rh, $^{115}$In | $^{103}$Rh, $^{115}$In | $^{103}$Rh, $^{115}$In |

**Table S3**: Major and trace element concentrations determined for Trace Elements Urine L-1

| Element | LOQ[a] | Trace Elements Urine L-1, LOT 1403080 | | |
|---|---|---|---|---|
| | $H_2$ mode / He mode | $H_2$ mode | He mode | Reference[b] |
| | **µg / ml** | **µg / ml** | **µg / ml** | **µg / ml** |
| $^{23}$Na | 0.1 / 0.1 | 2803 ± 81 | 2670 ± 137 | 2780[c, d] |
| $^{24}$Mg | 0.01 / 0.01 | 76.6 ± 4.2 | 79 ± 4 | 77.6 ± 17.4[d] |
| $^{39}$K | 0.1 / 0.3 | 1980 ± 92 | 1979 ± 195 | 1901[c, d] |
| $^{40}$Ca | 0.008 / - | 109 ± 4 | | 107[c, d] |
| $^{44}$Ca | - / 0.16 | | 114 ± 8 | 107[c, d] |
| | 'No gas' /He mode | 'No gas' mode | He mode | Reference[b] |
| | **ng / ml** | **ng / ml** | **ng / ml** | **ng / ml** |
| $^{59}$Co | 0.16 / 0.08 | 0.90 ± 0.17 | 0.78 ± 0.15 | 0.80 ± 0.16 |
| $^{63}$Cu | - / 0.7 | | 19.9 ± 3.5 | 20 ± 4 |
| $^{65}$Cu | 0.7 / - | 21.7 ± 4.0 | | 20 ± 4 |
| $^{66}$Zn | 0.3 / 0.15 | 365 ± 78 | 379 ± 79 | 347 ± 70 |
| $^{75}$As | - / 0.5 | | 151 ± 23 | 158 ± 32 |
| $^{85}$Rb | 0.6 / 0.6 | 1117 ± 136 | 1193 ± 163 | 1150[c] |
| $^{88}$Sr | 0.1 / 0.1 | 103 ± 13 | 104 ± 11 | 119[c] |
| $^{95}$Mo | 0.25 / 0.25 | 42.6 ± 2.7 | 40.3 ± 2.8 | 47.1 ± 9.5 |
| Pb[e] | 0.1 / 0.03 | 0.75 ± 0.18 | 0.71 ± 0.19 | 0.72 ± 0.36 |

The uncertainties of sample concentrations denote 1SD (standard deviation; n=2 for Na, Mg, K and Ca and n=5 for all other elements).

[a] LOQ = Limit of quantification (for He mode and $H_2$ mode or 'no gas' mode and He mode respectively).

[b] Reference data obtained using sector field (SF) ICP-MS and have expanded uncertainties with coverage factor k=2 at the 95 % confidence interval).[11]

[c] Additional approximate values, not certified

[d] Average of values determined by SF-ICP-MS and inductively coupled plasma optical emission spectroscopy (ICP-OES).

[e] Pb concentrations are calculated based on the average intensity of three lead isotopes: $^{206}$Pb, $^{207}$Pb and $^{208}$Pb.

**Table S4**: Major and trace element concentrations determined for bovine muscle ERM-BB184 and Human blood serum BCR-639.

| Isotope | Bovine muscle ERM-BB184 | | | | Human blood serum BCR-639 | | | |
|---|---|---|---|---|---|---|---|---|
| | LOQ[a] | This study n= | | Reference[b] | LOQ[a] | This study, n=2 | | Reference[b] |
| | 'No gas' / He mode | 'No gas' | He mode | | 'No gas' / He mode | 'No gas' | He mode | |
| | µg / g | | µg / g | µg / g | µg / ml | | µg / ml | |
| $^{23}$Na | - / 15 | | 1861 | 1804[c] | - / 4 | | 3454 ± 136 | |
| $^{24}$Mg | - / 8 | | 1060 | 997[c] | - / 0.25 | | 22.3 ± 0.3 | |
| $^{39}$K | - / 250 | | 15774 | 15844[c] | - / 7.2 | | 176 ± 37 | |
| $^{44}$Ca | - / 85 | | 257 | 155[c] | - / 2.4 | | 119 ± 15 | |
| | µg / g | | µg / g | µg / g | ng / ml | ng / ml | ng / ml | ng / ml |
| $^{59}$Co | 0.006 / 0.007 | 0.012 | 0.012 | | 0.8 / 0.8 | 1.20 ± 0.18 | 0.99 ± 0.06 | |
| $^{63}$Cu | - / 0.08 | | 2.29 | 2.31 ± 0.09 | - / 12 | | 1056 ± 10 | |
| $^{65}$Cu | 0.08 / 0.08 | 2.35 | 2.35 | 2.31 ± 0.09 | 12 / 12 | 1090 ± 16 | 1077 ± 12 | |
| $^{66}$Zn | 0.18 / 0.12 | 153 | 158 | 146 ± 7 | 26 / 17 | 2750 ± 3 | 3676 ± 25 | 2360 ± 140 |
| ID Zn[d] | | 151.5 ± 0.4[e] | | 146 ± 7 | | 2410 ± 100[e] | | 2360 ± 140 |
| $^{75}$As | - / 0.04 | | <0.04 | 0.020 ± 0.003 | - / 6 | | <6 | |
| $^{85}$Rb | 0.04 / 0.04 | 7.49 | 7.72 | | 6 / 6 | 157 ± 9 | 169 ± 14 | |
| $^{88}$Sr | 0.008 / 0.008 | 0.109 | 0.12 | | 1.2 / 1.2 | 45.2 ± 0.1 | 45.4 ± 1.6 | |
| $^{95}$Mo | - / 0.05 | | <0.05 | | - / 15 | | <15 | |
| Pb[f] | 0.003 / 0.003 | 0.011 | 0.01 | 0.0014-0.0158[g] | 0.4 / 0.4 | 2.77 ± 0.44 | 2.22 ± 0.24 | |

The uncertainties of sample concentrations (where shown) denote 1SD (standard deviation):

[a] LOQ = Limit of quantification

[b] Reference data have expanded uncertainties with coverage factor k=2 at the 95 % confidence interval[12,13]

[c] Additional values, not certified

[d] Determined by ID-MC-ICP-MS

[e] Values from Moore et al. 2017[14]

[f] Lead concentrations are calculated based on the average intensity of three lead isotopes: $^{206}$Pb, $^{207}$Pb and $^{208}$Pb.

[g] Not certified; Lead concentrations are likely to be non-homogenous in the sample

**Table S5**: Matrices of the correlation coefficients r and $r^2$ determined from the results of the 65 individual samples

| **r** | Na | Mg | K | Ca | Co | Cu | Zn | As | Rb | Sr | Mo |
|---|---|---|---|---|---|---|---|---|---|---|---|
| Na | - | | | | | | | | | | |
| Mg | 0.06 | - | | | | | | | | | |
| K | 0.48 | 0.11 | - | | | | | | | | |
| Ca | 0.09 | 0.29 | 0.00 | - | | | | | | | |
| Co | 0.02 | 0.37 | 0.14 | -0.45 | - | | | | | | |
| Cu | 0.12 | 0.20 | -0.02 | -0.15 | 0.12 | - | | | | | |
| Zn | -0.32 | -0.06 | -0.38 | 0.24 | -0.40 | 0.42 | - | | | | |
| As | -0.22 | 0.04 | -0.11 | 0.25 | -0.09 | -0.23 | 0.04 | - | | | |
| Rb | 0.37 | 0.13 | 0.90 | -0.13 | 0.21 | 0.01 | -0.33 | -0.16 | - | | |
| Sr | 0.24 | 0.39 | 0.12 | 0.81 | -0.23 | 0.04 | 0.23 | 0.01 | 0.04 | - | |
| Mo | 0.28 | 0.19 | 0.09 | -0.08 | 0.18 | 0.38 | -0.25 | -0.02 | 0.02 | -0.04 | - |
| Pb | 0.36 | 0.30 | 0.15 | 0.23 | 0.20 | 0.14 | 0.08 | 0.17 | 0.13 | 0.47 | 0.12 |

| **$r^2$** | Na | Mg | K | Ca | Co | Cu | Zn | As | Rb | Sr | Mo |
|---|---|---|---|---|---|---|---|---|---|---|---|
| Na | - | | | | | | | | | | |
| Mg | 0.00 | - | | | | | | | | | |
| K | 0.23 | 0.01 | - | | | | | | | | |
| Ca | 0.01 | 0.08 | 0.00 | - | | | | | | | |
| Co | 0.00 | 0.13 | 0.02 | 0.20 | - | | | | | | |
| Cu | 0.01 | 0.04 | 0.00 | 0.02 | 0.01 | - | | | | | |
| Zn | 0.10 | 0.00 | 0.15 | 0.06 | 0.16 | 0.18 | - | | | | |
| As | 0.05 | 0.00 | 0.01 | 0.06 | 0.01 | 0.05 | 0.00 | - | | | |
| Rb | 0.14 | 0.02 | 0.81 | 0.02 | 0.04 | 0.00 | 0.11 | 0.03 | - | | |
| Sr | 0.06 | 0.15 | 0.01 | 0.66 | 0.05 | 0.00 | 0.05 | 0.00 | 0.00 | - | |
| Mo | 0.08 | 0.04 | 0.01 | 0.01 | 0.03 | 0.14 | 0.06 | 0.00 | 0.00 | 0.00 | - |
| Pb | 0.13 | 0.09 | 0.02 | 0.05 | 0.04 | 0.02 | 0.01 | 0.03 | 0.02 | 0.22 | 0.01 |

White: low r and $r^2$, green: $0.4 < r < 0.5$, $0.2 < r^2 < 0.8$, red: $r > 0.8$, $r^2 > 0.65$.

**Table S6:** Daily mean urinary element concentrations of the study participants P1 to P10.

| Participant (n - d) | Na µg/ml | Mg µg/ml | K µg/ml | Ca µg/ml | Co ng/ml | Cu ng/ml | Zn ng/ml | As ng/ml | Rb ng/ml | Sr ng/ml | Mo ng/ml | Pb ng/ml |
|---|---|---|---|---|---|---|---|---|---|---|---|---|
| P1 (8 - 2) | 3351 | 98.5 | 3655 | 220 | 0.20 | 9.22 | 530 | 82.5 | 2143 | 177 | 27.3 | 0.73 |
| P2 (6 - 2) | 5535 | 112 | 5132 | 139 | 0.42 | 10.1 | 202 | 42.8 | 2052 | 183 | 64.8 | 0.97 |
| P3 (5 - 1) | 1466 | 117 | 3519 | 213 | 0.41 | 10.1 | 318 | 187 | 1352 | 159 | 41.8 | 0.74 |
| P4 (9 - 2) | 3320 | 114 | 4654 | 158 | 0.54 | 12.5 | 220 | 75.6 | 1950 | 124 | 92.4 | 0.46 |
| P5 (4 - 1) | 5318 | 161 | 6489 | 132 | 1.83 | 12.6 | 96.4 | 3.52 | 2821 | 229 | 95.1 | 0.96 |
| P6 (8 - 2) | 2395 | 110 | 4167 | 16.9 | 1.56 | 10.4 | 134 | 22.0 | 2203 | 58.4 | 31.6 | 0.61 |
| P7 (9 - 2) | 3265 | 98.5 | 3222 | 217 | 0.14 | 8.89 | 300 | 5.68 | 1722 | 231 | 32.7 | 0.60 |
| P8 (4 - 1) | 5958 | 60.5 | 2499 | 104 | 0.25 | 14.6 | 468 | 3.73 | 1446 | 109 | 66.4 | 0.66 |
| P9 (7 - 2) | 2028 | 62.7 | 2235 | 117 | 0.17 | 14.7 | 683 | 9.41 | 1244 | 143 | 41.3 | 0.54 |
| P10 (5 - 1) | 4144 | 134 | 5989 | 181 | 0.21 | 13.7 | 414 | 7.46 | 2255 | 239 | 64.3 | 0.76 |
| Mean | 3678 | 107 | 4156 | 150 | 0.57 | 11.7 | 336 | 43.9 | 1919 | 165 | 55.8 | 0.70 |
| Median | 3335 | 111 | 3911 | 148 | 0.33 | 11.4 | 309 | 15.7 | 2001 | 168 | 53.1 | 0.69 |
| SD | 1457 | 29 | 1340 | 60 | 0.58 | 2.1 | 177 | 55.3 | 460 | 56 | 23.4 | 0.16 |
| CV (%) | 40 | 27 | 32 | 40 | 101 | 18 | 53 | 126 | 24 | 34 | 42 | 23 |

Participants: n = number of samples; d = length of sampling period in days.

Deviation from the median value for all participants by a factor of:
1-0.75 and 1-1.25 (white)
0.76-0.50 and 1.26-1.50 (green)
0.49-0.25 and 1.51-2.00 (yellow)
<0.25 and >2 (red)

**Table S7a**: Coefficients of variation (CV values in %) for element concentrations of urine samples collected on a single day for the study participants P1 to P10.

| CV values (%) | Na | Mg | K | Ca | Co | Cu | Zn | As | Rb | Sr | Mo | Pb |
|---|---|---|---|---|---|---|---|---|---|---|---|---|
| P1 - Day 1 | 21 | 11 | 34 | 21 | 20 | 18 | 22 | 19 | 31 | 14 | 39 | 12 |
| P1 - Day 2 | 53 | 37 | 75 | 58 | 27 | 12 | 31 | 55 | 76 | 40 | 21 | 15 |
| P2 - Day 1 | 6 | 7 | 41 | 32 | 21 | 15 | 47 | 11 | 25 | 29 | 56 | 9 |
| P2 - Day 2 | 15 | 61 | 33 | 15 | 40 | 25 | 91 | 21 | 28 | 14 | 43 | 17 |
| P3 | 14 | 22 | 79 | 39 | 49 | 38 | 40 | 102 | 54 | 26 | 34 | 33 |
| P4 - Day 1 | 55 | 114 | 59 | 19 | 45 | 19 | 27 | 19 | 22 | 16 | 6 | 19 |
| P4 - Day 2 | 36 | 50 | 68 | 64 | 31 | 19 | 48 | 16 | 52 | 48 | 39 | 26 |
| P5 | 46 | 78 | 69 | 81 | 15 | 38 | 27 | 115 | 63 | 65 | 19 | 26 |
| P6 - Day 1 | 20 | 47 | 58 | 71 | 30 | 6 | 43 | 26 | 46 | 59 | 15 | 7 |
| P6 - Day 2 | 18 | 24 | 44 | 31 | 38 | 21 | 49 | 19 | 35 | 26 | 41 | 18 |
| P7 - Day 1 | 22 | 26 | 49 | 20 | 25 | 15 | 48 | 18 | 30 | 9 | 51 | 8 |
| P7 - Day 2 | 23 | 24 | 61 | 19 | 41 | 8 | 26 | 58 | 47 | 17 | 30 | 18 |
| P8 | 31 | 14 | 17 | 10 | 50 | 9 | 37 | 4 | 14 | 8 | 32 | 23 |
| P9 - Day 1 | 40 | 31 | 14 | 71 | 18 | 19 | 57 | 25 | 27 | 55 | 26 | 50 |
| P9 - Day 2 | 57 | 42 | 31 | 38 | 5 | 14 | 31 | 30 | 17 | 28 | 23 | 19 |
| P10 | 9 | 16 | 30 | 22 | 22 | 12 | 27 | 24 | 30 | 18 | 43 | 16 |
| 1-Day Mean | 29 | 38 | 48 | 38 | 30 | 18 | 41 | 35 | 37 | 29 | 32 | 20 |

CV values marked green are >25% and ≤50%; yellow CVs are >50% and ≤100%; red CVs are >100%.

**Table S7b**: Coefficients of variation (CV values in %) for element concentrations of urine samples collected on two non-consecutive days for six study participants.

| CV values (%) | Na | Mg | K | Ca | Co | Cu | Zn | As | Rb | Sr | Mo | Pb |
|---|---|---|---|---|---|---|---|---|---|---|---|---|
| P1 - Days 1+2 | 39 | 27 | 54 | 45 | 29 | 16 | 29 | 115 | 52 | 33 | 38 | 25 |
| P2 - Days 1+2 | 11 | 41 | 34 | 38 | 31 | 21 | 62 | 21 | 25 | 28 | 53 | 18 |
| P4 - Days 1+2 | 45 | 72 | 62 | 48 | 44 | 20 | 37 | 65 | 39 | 36 | 28 | 24 |
| P6 - Days 1+2 | 26 | 35 | 48 | 50 | 33 | 15 | 43 | 58 | 38 | 43 | 28 | 13 |
| P7 - D1+2 | 22 | 23 | 59 | 18 | 42 | 15 | 43 | 59 | 44 | 17 | 44 | 19 |
| P9 - Days 1+2 | 98 | 46 | 52 | 68 | 15 | 16 | 78 | 81 | 43 | 49 | 95 | 37 |
| 2-Day Mean | 40 | 41 | 52 | 45 | 32 | 17 | 49 | 67 | 40 | 34 | 48 | 23 |
| 2-Day/1-Day | 1.4 | 1.1 | 1.1 | 1.2 | 1.1 | 0.9 | 1.2 | 1.9 | 1.1 | 1.2 | 1.5 | 1.2 |

CV values marked green are >25% and ≤50%; yellow CVs are >50% and ≤100%; red CVs are >100%.

**Table S8**. Z-scores for the first void samples collected from participants P1 to P10 on one or two days

| z-score | Na | Mg | K | Ca | Co | Cu | Zn | As | Rb | Sr | Mo | Pb |
|---|---|---|---|---|---|---|---|---|---|---|---|---|
| P1 - Day 1 | 0.9 | 1.0 | 1.2 | 0.4 | 1.2 | 0.8 | 1.4 | 0.3 | 1.1 | 0.3 | 0.4 | 0.3 |
| P1 - Day 2 | 0.3 | 1.0 | 0.7 | 0.4 | 0.9 | 0.2 | 0.2 | 1.4 | 0.7 | 0.7 | 1.5 | 0.6 |
| P2 - Day 1 | 0.7 | 0.5 | 1.1 | 0.8 | 0.9 | 1.1 | 1.0 | 1.1 | 1.1 | 1.0 | 0.9 | 1.1 |
| P2 - Day 2 | 1.0 | 1.1 | 1.1 | 1.0 | 1.2 | 1.2 | 1.1 | 1.1 | 1.2 | 1.1 | 1.1 | 1.1 |
| P3 | 1.0 | 0.7 | 0.3 | 1.1 | 1.2 | 0.1 | 0.7 | 0.4 | 0.1 | 0.9 | 1.1 | 0.5 |
| P4 - Day 1 | 1.1 | 1.2 | 0.8 | 0.7 | 1.2 | 0.6 | 0.9 | 0.6 | 0.6 | 1.0 | 0.3 | 1.5 |
| P4 - Day 2 | 0.3 | 0.0 | 0.6 | 0.4 | 0.4 | 0.5 | 0.0 | 0.9 | 0.6 | 0.3 | 0.2 | 0.4 |
| P5 | 2.6 | 0.5 | 11.1 | 0.3 | 0.0 | 0.0 | 0.7 | 0.0 | 9.1 | 0.7 | 0.4 | 0.0 |
| P6 - Day 1 | 0.0 | 1.5 | 1.2 | 1.5 | 1.1 | 1.1 | 1.5 | 1.4 | 1.3 | 1.5 | 1.5 | 1.0 |
| P6 - Day 2 | 0.6 | 0.8 | 0.9 | 0.6 | 1.4 | 1.1 | 0.6 | 1.4 | 0.7 | 0.5 | 0.3 | 0.3 |
| P7 - Day 1 | 0.9 | 0.4 | 0.9 | 0.5 | 0.7 | 0.6 | 0.0 | 1.2 | 0.8 | 0.5 | 0.2 | 1.4 |
| P7 - Day 2 | 1.9 | 0.7 | 1.5 | 1.7 | 1.5 | 1.3 | 4.8 | 2.1 | 1.0 | 0.3 | 1.5 | 2.7 |
| P8 | 0.1 | 0.5 | 2.0 | 1.1 | 0.4 | 2.1 | 0.4 | 0.5 | 2.3 | 2.1 | 2.2 | 0.5 |
| P9 - Day 1 | 0.8 | 1.1 | 0.4 | 0.9 | 0.3 | 0.7 | 0.3 | 0.0 | 0.0 | 1.0 | 1.5 | 0.2 |
| P9 - Day 2 | 1.0 | 0.5 | 0.8 | 0.8 | 0.7 | 0.4 | 0.9 | 0.9 | 0.7 | 0.9 | 0.1 | 1.1 |
| P10 | 1.4 | 1.7 | 1.1 | 1.3 | 0.6 | 0.3 | 0.8 | 1.5 | 1.0 | 1.6 | 0.3 | 0.3 |
| Mean z-score | 0.9 | 0.8 | 1.6 | 0.8 | 0.9 | 0.8 | 1.0 | 0.9 | 1.4 | 0.9 | 0.8 | 0.8 |

White: Z-score ≤ 0.5, green: z-score = 0.6-1.0, yellow: z-score = 1.1-2.0, red: z-score >2.

**Table S9a.** Mean urinary element concentrations of participant groups that differ in their smoking status.

| Smoking status | n | Na µg/ml | Mg µg/ml | K µg/ml | Ca µg/ml | Co ng/ml | Cu ng/ml | Zn ng/ml | As ng/ml | Rb ng/ml | Sr ng/ml | Mo ng/ml | Pb ng/ml |
|---|---|---|---|---|---|---|---|---|---|---|---|---|---|
| Current | 1 | 5318 | 161 | 6489 | 132 | 1.83 | 12.6 | 96 | 3.5 | 2821 | 229 | 95 | 0.96 |
| Occasional | 1 | 5535 | 112 | 5132 | 139 | 0.42 | 10.1 | 202 | 42.8 | 2052 | 183 | 65 | 0.97 |
| Past | 2 | 2830 | 104 | 3694.5 | 117 | 0.85 | 9.7 | 217 | 13.9 | 1963 | 145 | 33 | 0.61 |
| Never | 6 | 3378 | 98 | 3759 | 166 | 0.30 | 12.5 | 439 | 60.9 | 1732 | 159 | 55 | 0.65 |
| Mean | 10 | 3678 | 107 | 4156 | 150 | 0.57 | 11.7 | 337 | 43.9 | 1919 | 165 | 56 | 0.70 |

The mean group values are calculated from the mean values of n individuals in the respective group. n = number of participants.

**Table S9b.** Mean urinary element concentrations of participant groups that differ in their alcohol intake.

| Alcohol intake | n | Na µg/ml | Mg µg/ml | K µg/ml | Ca µg/ml | Co ng/ml | Cu ng/ml | Zn ng/ml | As ng/ml | Rb ng/ml | Sr ng/ml | Mo ng/ml | Pb ng/ml |
|---|---|---|---|---|---|---|---|---|---|---|---|---|---|
| ≥3 u/d | 2 | 4839.5 | 123 | 5561 | 160 | 0.32 | 11.9 | 308 | 25.2 | 2154 | 211 | 64.5 | 0.87 |
| 1-2 u/d | 2 | 2211.5 | 86.6 | 3201 | 67.0 | 0.87 | 12.6 | 409 | 15.7 | 1724 | 101 | 36.5 | 0.58 |
| < 1 u/d | 6 | 3780 | 108.4 | 4006 | 174 | 0.6 | 11.3 | 322 | 59.6 | 1906 | 172 | 59 | 0.69 |
| Mean | 10 | 3678 | 107 | 4156 | 149.8 | 0.57 | 11.7 | 337 | 43.9 | 1919 | 165 | 55.7 | 0.70 |

The mean group values are calculated from the mean values of n individuals in the respective group. n = number of participants, u/d = units*/day
*Units denote UK Alcohol units

**Table S9c.** Mean urinary element concentrations of participant groups that differ in their caffeine intake.

| Caffeine intake | n | Na µg/ml | Mg µg/ml | K µg/ml | Ca µg/ml | Co ng/ml | Cu ng/ml | Zn ng/ml | As ng/ml | Rb ng/ml | Sr ng/ml | Mo ng/ml | Pb ng/ml |
|---|---|---|---|---|---|---|---|---|---|---|---|---|---|
| 3+ d/d | 5 | 3810 | 98.7 | 3809 | 118 | 0.80 | 12.3 | 382 | 24.2 | 1971 | 143 | 52.2 | 0.70 |
| 1-2 d/d | 3 | 3715 | 121 | 4880 | 178 | 0.35 | 11.3 | 311 | 78.9 | 1886 | 194 | 57.0 | 0.82 |
| Rarely | 2 | 3293 | 106 | 3938 | 188 | 0.34 | 10.7 | 260 | 40.7 | 1836 | 178 | 62.5 | 0.53 |
| Mean | 10 | 3678 | 107 | 4156 | 150 | 0.57 | 11.7 | 337 | 43.9 | 1919 | 165 | 55.7 | 0.70 |

The mean group values are calculated from the mean values of n individuals in the respective group. n = number of participants, d/d = drinks*/day
*Caffeine drinks include coffee, tea and caffeinated soft drinks.

**Table S10**. Data to evaluate possible relationships of urinary element concentrations with gender.

| | Gender | Na µg/ml | Mg µg/ml | K µg/ml | Ca µg/ml | Co ng/ml | Cu ng/ml | Zn ng/ml | As ng/ml | Rb ng/ml | Sr ng/ml | Mo ng/ml | Pb ng/ml |
|---|---|---|---|---|---|---|---|---|---|---|---|---|---|
| Mean | female | 3606 | 114 | 4539 | 141 | 0.76 | 10.7 | 259 | 64.6 | 2088 | 147 | 56.9 | 0.72 |
| Median | female | 3357 | 111 | 4270 | 158 | 0.44 | 10.4 | 221 | 36.4 | 2072 | 152 | 46.5 | 0.68 |
| SD | female | 1469 | 28 | 1031 | 77 | 0.63 | 1.4 | 158 | 65.7 | 383 | 59 | 29.3 | 0.22 |
| CV (%) | female | 41 | 25 | 23 | 55 | 83 | 14 | 61 | 102 | 18 | 40 | 52 | 30 |
| Male | male | 3512 | 85 | 3255 | 157 | 0.18 | 12.5 | 458 | 7.3 | 1616 | 181 | 47.8 | 0.61 |
| Median | male | 3540 | 88 | 2915 | 162 | 0.18 | 14.1 | 383 | 5.6 | 1603 | 184 | 52.4 | 0.60 |
| SD | male | 1677 | 33 | 1562 | 57 | 0.05 | 3.0 | 264 | 5.2 | 492 | 61 | 27.4 | 0.09 |
| CV (%) | male | 48 | 39 | 48 | 36 | 27 | 24 | 58 | 71 | 30 | 34 | 57 | 15 |
| Mean/Mean | male/fem | 1.0 | 0.7 | 0.7 | 1.1 | 0.2 | 1.2 | 1.8 | 0.1 | 0.8 | 1.2 | 0.8 | 0.8 |
| Median/Median | male/fem | 1.1 | 0.8 | 0.7 | 1.0 | 0.4 | 1.4 | 1.7 | 0.2 | 0.8 | 1.2 | 1.1 | 0.9 |
| P | male vs. fem | 0.91 | 0.09 | 0.07 | 0.67 | 0.04 | 0.11 | 0.08 | 0.05 | 0.05 | 0.29 | 0.55 | 0.26 |

Results that may be indicative of significant differences between genders are highlighted in red.

**Table S11:** Data to evaluate possible relationships of urinary element concentrations with diet groups.

| | Diet group | Na µg/ml | Mg µg/ml | K µg/ml | Ca µg/ml | Co ng/ml | Cu ng/ml | Zn ng/ml | As ng/ml | Rb ng/ml | Sr ng/ml | Mo ng/ml | Pb ng/ml |
|---|---|---|---|---|---|---|---|---|---|---|---|---|---|
| Mean | omv | 3372 | 88.4 | 3370 | 169 | 0.26 | 11.62 | 427 | 40.3 | 1737 | 161 | 51.3 | 0.59 |
| Median | omv | 3387 | 85.7 | 3310 | 163 | 0.19 | 11.28 | 352 | 10.8 | 1888 | 157 | 40.5 | 0.55 |
| SD | omv | 1344 | 30.4 | 1180 | 54 | 0.17 | 2.74 | 241 | 57.6 | 445 | 49 | 32.0 | 0.13 |
| CV (%) | omv | 40 | 34 | 35 | 32 | 67 | 24 | 56 | 143 | 26 | 30 | 62 | 22 |
| Mean | no-m | 4400 | 127.4 | 5252 | 161 | 0.66 | 11.30 | 246 | 56.6 | 2107 | 199 | 66.2 | 0.88 |
| Median | no-m | 5318 | 134.5 | 5489 | 180 | 0.41 | 11.13 | 233 | 36.5 | 2181 | 217 | 64.3 | 0.85 |
| SD | no-m | 1742 | 29.4 | 1158 | 46 | 0.66 | 1.88 | 124 | 75.1 | 534 | 42 | 21.2 | 0.15 |
| CV (%) | no-m | 40 | 23 | 22 | 28 | 101 | 17 | 50 | 133 | 25 | 21 | 32 | 17 |
| Mean/Mean-omv | no-m | 1.3 | 1.4 | 1.6 | 1.0 | 2.5 | 1.0 | 0.6 | 1.4 | 1.2 | 1.2 | 1.3 | 1.5 |
| Median/Median-omv | no-m | 1.6 | 1.6 | 1.7 | 1.1 | 2.2 | 1.0 | 0.7 | 3.4 | 1.2 | 1.4 | 1.6 | 1.6 |
| P (vs. omv) | no-m | 0.24 | 0.04 | 0.01 | 0.79 | 0.10 | 0.82 | 0.15 | 0.66 | 0.19 | 0.17 | 0.37 | 0.003 |
| Mean | no-d | 3369 | 127.3 | 4941 | 55 | 1.65 | 11.14 | 122 | 15.8 | 2409 | 115 | 52.7 | 0.72 |
| Median | no-d | 2820 | 111.0 | 4323 | 21 | 1.66 | 10.51 | 131 | 11.4 | 2235 | 70 | 33.0 | 0.62 |
| SD | no-d | 1740 | 29.3 | 1350 | 67 | 0.19 | 1.23 | 22 | 15.0 | 358 | 99 | 36.7 | 0.21 |
| CV (%) | no-d | 52 | 23 | 27 | 121 | 11 | 11 | 18 | 95 | 15 | 86 | 70 | 29 |
| Mean/Mean-omv | no-d | 1.0 | 1.4 | 1.5 | 0.3 | 6.4 | 1.0 | 0.3 | 0.4 | 1.4 | 0.7 | 1.0 | 1.2 |
| Median/Median-omv | no-d | 0.8 | 1.3 | 1.3 | 0.1 | 8.8 | 0.9 | 0.4 | 1.1 | 1.2 | 0.4 | 0.8 | 1.1 |
| P (vs. omv) | no-d | 1.00 | 0.08 | 0.08 | 0.01 | 3.1E-07 | 0.78 | 0.06 | 0.50 | 0.04 | 0.30 | 0.95 | 0.20 |

Results that may be indicative of significant differences between diet groups are highlighted in red.
omv = omnivore, no-m = no meat, no-d = no dairy.

## References


1. USDA Food Composition Databases, Nutrient Lists, United States Department of Agriculture (ndb.nal.gov).

2. Enig, M. G., 2010, Mineral Nutrients. In *Kirk-Othmer Encyclopedia of Chemical Technology*.

3. NHS, Vitamins and minerals, (nhs.uk/conditions/vitamins-and-minerals/others).

4. Cabrera, C., Lloris, F., et al. 2003, Mineral content in legumes and nuts: contribution to the Spanish dietary intake. *Sci. Total Environ*. **308**, 1-14.

5. World Health Organisation (ww.who.int).

6. European Food Safety Authority, 2014. Dietary exposure to inorganic arsenic in the European population, *EFSA Journal*, **12, 3597**.

7. Anke, M., Angelow, L., 1995, Rubidium in the food chain, *Fresenius' J. Anal. Chem.* **352**, 236-239.

8. Schroeder, H. A., Tipon, I. H., Nason, A. P., 1972. Trace metals in man: Strontium and barium, *J. Chron. Dis.* **25**, 491-517.

9. European Food Safety Authority, 2012. Lead dietary exposure in the European population, *EFSA J.* **10**, 2831**.**

10. Guérin, T, Le Calvez et al. 2017, Levels of lead in foods from the first French total diet study on infants and toddlers. *Food Chem.* **237**, 849-856.

11. Seronorm, Seronorm ™ Trace Elements Urine L-1, Lot 1403080, 2015.

12. J. Snell and J. Charoud-Got. Certification Report. The Certification of the Mass Fractions of Elements in Bovine Muscle Certified Reference Material ERM®-BB184, *EUR 25481 EN – 2012*, European Union, 2012.

13. Institute for Reference Materials and Measurements (IRMM), *Certified Reference Material BCR-639*, 2007, https://ec.europa.eu/jrc/sites/default/files/rm/BCR-639_cert.pdf

14. R. E. T. Moore, F. Larner, B. J. Coles and M. Rehkämper. High precision zinc stable isotope measurement of certified biological reference materials using the Double Spike Technique and Multiple Collector-ICP-MS, *Anal. Bioanal. Chem*., 2017, **409**, 2941-2950.
